# Walking Distance and Interlimb Gait Differences Across 51 kg of Weight Loss After Total Knee Arthroplasty: A Three-Session Wearable Sensor Case Report

**DOI:** 10.3390/reports9040345

**Published:** 2026-10-12

**Authors:** Andrei Machado Viegas da Trindade, Leonardo Pinheiro Rezende, Junichiro Sado Junior, Marcos Henrique Alves Resende, Claudia Santos Oliveira

**Affiliations:** 1 Faculty of Medicine, University Evangelica of Goiás, Anápolis 75083-515, Brazil; 2 Department of Orthopedics Surgery, Hospital Municipal de Aparecida de Goiânia (HMAP), Aparecida de Goiânia 74936-600, Brazil; 3 Postgraduate Program in Health Sciences, Santa Casa de São Paulo School of Medical Sciences, São Paulo 01221-020, Brazil; 4 Hospital Israelita Albert Einstein, São Paulo 05652-900, Brazil; 5 Human Movement and Rehabilitation Post Graduation Program, University Evangelica of Goiás, Anápolis 75083-515, Brazil; 6 Health Sciences Program, Santa Casa de São Paulo School of Medical Sciences, São Paulo 01221-020, Brazil

**Keywords:** total knee arthroplasty, bariatric surgery, weight loss, gait analysis, wearable inertial sensor, interlimb asymmetry, case report

## Abstract

**Background and Clinical Significance:** Longitudinal observations that combine major postoperative weight loss with limb-specific gait assessment after total knee arthroplasty (TKA) are uncommon. This case report describes three single-trial instrumented assessments in one patient and is intended as a descriptive observation; it does not establish causality, measurement stability, mechanical loading, or a clinically meaningful persistent deficit; **Case Presentation:** A man in his early sixties at the first assessment had undergone left TKA for end-stage osteoarthritis and subsequently underwent Roux-en-Y gastric bypass. Three assessments were available from approximately 31 to 67 months after TKA. Body mass was 120, 88, and 69 kg (body mass index 43.03, 31.55, and 24.74 kg/m^2^), while two-minute walk distance (2MWT) was 67.8, 105.1, and 118.1 m. The third walk test was launched in the software’s six-minute module and stopped at 1 min 58 s, so 118.1 m is the distance accumulated over 118 s and was neither adjusted nor extrapolated to 120 s. The operated limb showed longer stance duration than the non-operated limb by 3.04, 5.28, and 2.05 percentage points, and a proprietary propulsion index was lower on the operated side by 18.18%, 17.95%, and 14.06%; the direction of both differences was the same at every session but the magnitude was not, and the index has no published calculation or post-TKA interpretive threshold. Each session comprised one trial, and the third-session gait means were based on only five operated-limb and four non-operated-limb steps. Valid Timed Up and Go (TUG) totals were available for sessions 1 and 2 (13.41 and 14.95 s); the available source documentation did not contain a valid session-3 TUG result. Vendor-derived walk-test speed was excluded from primary interpretation because its calculation could not be reconciled with distance divided by test duration; **Conclusions:** Across these three observations, 2MWT distance increased while side-to-side differences in stance duration and the exploratory propulsion index were present at each session. These co-occurring changes cannot be attributed to weight loss specifically, because time since arthroplasty, rehabilitation exposure and repeated testing varied together with body mass. The findings are descriptive and hypothesis-generating; confirmation requires prospective studies with repeated trials per session, an adequate number of analysed steps, and validated kinetic or limb-specific outcomes.

## 1. Introduction and Clinical Significance

Total knee arthroplasty (TKA) is among the most frequently performed elective orthopaedic procedures, and demand continues to rise [1,2]. It is undertaken at the end stage of a progressive joint disease in which structural deterioration, altered joint loading and inflammatory processes interact, and for which conservative and biologic treatments have not been shown to reverse established damage [3]. Many candidates have obesity, which alters lower-limb walking mechanics and is associated with less favourable postoperative trajectories [4,5,6]. Limb-to-limb differences in stance, single-limb support and propulsive performance have been reported after TKA even when pain and patient-reported outcomes improve [7,8,9,10].

Bariatric surgery produces large, sustained weight loss and is increasingly undertaken by patients who have had, or will have, joint replacement [11,12]. After substantial weight loss, walking kinematics and whole-task performance may change [13,14,15,16]. Whether whole-task improvements are accompanied by parallel changes in limb-specific gait measures after TKA is less well documented, and whole-body and joint-level status are already known to diverge deficient proximal trunk stability has been associated with radiographic progression of knee osteoarthritis independently of the joint itself [17].

Wearable inertial sensors can quantify spatiotemporal gait variables outside a motion-analysis laboratory and are increasingly used after TKA [18,19,20]. Whole-test distance and limb-specific spatiotemporal variables answer different questions. The first summarises how far a patient can walk when the task is performed as a whole; the second describes how the two limbs divide that task between them. Assessing them together is what allows a study to test whether a gain in walking capacity is accompanied by a corresponding reduction in interlimb difference, or whether the two can move independently of each other. We therefore describe three instrumented assessments in one patient spanning a 51 kg reduction in body mass after TKA and bariatric surgery. The report focuses on directly reported spatiotemporal variables and a proprietary propulsion index, while separating observed numerical differences from claims about measurement stability, mechanical loading or clinical importance.

Clinical Significance: Whole-test walking distance and limb-specific gait variables describe different aspects of function, in the same way that functional and other domains of recovery after TKA are only partly correlated [21]. In this patient the two did not convey the same message, and because each session comprised a single trial the interlimb differences should not be read as proof of a stable or clinically meaningful deficit.

## 2. Case Presentation

The case is reported using the CAse REport (CARE) guideline framework [22]. When information was absent from the source documentation, it is stated as not recorded rather than inferred. A completed CARE checklist, the acquisition protocol, clarifies the status of proprietary device outputs, and documentation of which outcomes were considered valid for longitudinal reporting are provided as Supplementary Materials S1–S4.

### 2.1. Ethics, Consent and Patient Perspective

The patient provided written informed consent for publication of this case report and the accompanying anonymised data and figures, and the work was conducted in accordance with the Declaration of Helsinki. The assessments were performed as part of routine care and of an institutional research protocol approved by the Research Ethics Committee (Comitê de Ética em Pesquisa) of the Universidade Evangélica de Goiás, Anápolis, Goiás, Brazil, under CAAE 52052421.9.0000.5076; the currently approved version of that protocol received opinion number 6,775,127 on 19 April 2024, in accordance with Brazilian National Health Council Resolution 466/2012. According to the host institution, the present de-identified single-patient report did not require additional ethics-committee review. A structured patient perspective was not collected at the time of assessment and could not be obtained retrospectively without breaching the de-identification conditions under which the data were released.

### 2.2. Patient Information and Clinical Findings

At the first assessment, the patient was a man in his early sixties with class III obesity and end-stage primary osteoarthritis of the left knee. Height was 1.67 m, body mass was 120 kg, and body mass index (BMI) was 43.03 kg/m^2^. He walked independently without an assistive device at all three assessments. The available documentation did not record presenting symptoms, detailed physical examination, radiographic osteoarthritis grade, coronal deformity, range of motion, prosthesis alignment, radiographic status at follow-up, comorbidities, medication, nutritional status, smoking history or occupation. The indication for TKA was documented as end-stage osteoarthritis. A Western Ontario and McMaster Universities Osteoarthritis Index (WOMAC) was available only at the first session (pain 1 of 20, stiffness 0 of 8, physical function 11 of 68, total 12 of 96), so no longitudinal patient-reported change can be described. The source records were re-examined for the present revision. Beyond these session-1 WOMAC subscale scores and the documented absence of an assistive device at every session, they contained no entries on interval knee pain, functional complaints, postoperative complications or rehabilitation characteristics, and no such information could be reconstructed retrospectively.

### 2.3. Timeline and Interventions

Left TKA was performed at month 0 through a subvastus approach using a rotating-platform implant. The manufacturer, model, bearing material, fixation, patellar management, operative time and alignment strategy were not recorded. The first instrumented assessment occurred at approximately 31 months after TKA and preceded bariatric surgery. A Roux-en-Y gastric bypass was performed between the first and second sessions, but the exact date was not recorded. The second assessment occurred during the following calendar year, and the third occurred at approximately 67 months after TKA, with the latter timing fixed by the instrument reports. Exact collection dates for sessions 1 and 2 were unavailable. Because these dates are missing, the interval between sessions 1 and 2 and the position of the bariatric procedure within that interval are both indeterminate, and the 51 kg reduction is known only as a total across the observation period. Postoperative rehabilitation was prescribed after both procedures, but its type, frequency, duration, adherence and associated adverse events were not recorded.

### 2.4. Diagnostic Assessment

Gait was assessed with a single wireless inertial measurement unit (IMU; G-Walk, BTS Bioengineering, Garbagnate Milanese, Italy). The sensor was secured over the L5 spinous process with the manufacturer’s elastic belt and sampled at 100 Hz. Data were processed with the manufacturer’s G-Studio software, by the same assessor at each session.

The acquisition used the vendor default protocols for the Walk, TUG and walk-test modules. No user-configurable processing setting was altered at any session, the automatic segmentation produced by the software was accepted as exported, and no trial was rejected or repeated. Within that pipeline, the filter type and cut-off frequencies, the gait-event detection rule, the trial-rejection criteria and the acceleration and deceleration exclusion windows are neither user-accessible nor published by the manufacturer. The software and firmware version strings were not printed on the archived instrument reports and were not recorded separately at the time of assessment. Exact reproduction of the signal processing is therefore not possible, and the values reported here should be read as the output of one commercial implementation rather than of a transparent algorithm. Independent validation has been reported for several spatiotemporal outputs of trunk-mounted systems in other populations [23,24], but that evidence does not validate the proprietary composite indices used by this device after TKA, and the wider difficulty of validating kinematic algorithms embedded in portable, low-cost inertial hardware has been documented previously [25].

Each session comprised one straight-line instrumented walk, one TUG and one walk test. Straight-line walk analysis durations were 29.4, 26.3 and 30.2 s. The software retained 10, 9 and 5 operated-limb steps and 10, 10 and 4 non-operated-limb steps at sessions 1 to 3. The length of the walking course, the way turns were performed, and whether an identical course configuration was used at all three sessions were not recorded in the source documentation and cannot be verified retrospectively. The count of completed course lengths reported by the software is an internal device output from which the course geometry cannot be recovered. Comparisons of walk-test distance across sessions should be read with this uncertainty in mind.

The number of gait cycles available constrains what these data can describe. Nine to ten steps per limb at sessions 1 and 2, and five operated-limb and four non-operated-limb steps at session 3, fall below the step counts reported to be necessary for stable kinetic, kinematic and gait-variability averages in instrumented locomotion protocols, where thresholds on the order of several tens of strides have been proposed [26]. No equivalent threshold has been established for a two-minute overground protocol in a post-arthroplasty patient, so the comparison indicates the scale of the shortfall rather than a formal criterion. The direction is nevertheless unambiguous: the session-3 means describe the few steps the software retained and cannot be taken as a stable description of this patient’s habitual gait pattern, and the sessions 1 and 2 means, while better supported, remain single-trial estimates. Reported standard deviations accordingly describe step-to-step dispersion within a single trial; they are not measures of test–retest reliability, and repeated steps from one individual are not independent observations.

No inferential statistics were applied at any stage, and this was a design decision rather than an omission. With one patient, one trial per session and steps drawn from within that single trial, the repeated observations do not constitute an independent sampling distribution; a p value or confidence interval computed from them would describe dispersion among steps of one walk while appearing to describe the precision of a population estimate. Every comparison reported here is therefore a numerical description of single observations.

Numerical convention: values computed by the authors, including body mass index, percentage-point differences and relative differences, are reported to two decimal places throughout the text, tables and figures. Values exported directly by the instrument are reported at the precision at which the software exported them and are not padded to a precision the device did not provide.

The walk test was administered as a two-minute walk test (2MWT) at all sessions. At session 3 the operator launched the protocol in the software’s six-minute module and terminated it at 1 min 58 s. The reported value of 118.1 m is therefore the distance accumulated over 118 s as exported by the software. No adjustment, pro-rating or extrapolation to 120 s was applied at any stage; for reference, simple pro-rating would have given 120.10 m, and that figure is not used anywhere in this report. The session-3 distance is thus derived from 2 s less walking than sessions 1 and 2 and is flagged accordingly wherever it is reported. No rest intervals were taken.

The device also produced a proprietary walk-test speed output that was not equal to distance divided by total test duration. The vendor does not disclose the calculation or whether turning and other non-steady-state intervals are excluded. Because its derivation is unknown, this output is reported only in the Supplementary Material and is not interpreted as conventional whole-test walking speed.

Vendor-reported walk-test cadence and stride length are retained on a different basis from the speed output. Both are direct per-step quantities produced by the gait-event detection whose spatiotemporal accuracy has been examined independently in trunk-mounted systems [23,24], whereas the speed output is a composite the vendor presents as a whole-test quantity. An internal check supports the distinction. Multiplying the reported cadence by the reported stride length gives 86.10, 132.44 and 149.45 m for the three sessions, which exceeds the measured walk-test distance by 27.00%, 26.01% and 26.55%, a near-constant factor across sessions that differed widely in distance covered. That pattern is what would be expected if cadence and stride length describe the straight-line, steady-state portions of the course while excluding turns and the accelerations and decelerations around them: internally coherent for per-step descriptors, but not reconcilable with a whole-test average that necessarily includes the turns. Cadence and stride length are therefore reported as descriptors of detected steady-state walking, are labelled as such where they are tabulated, and are not used to reconstruct walk-test distance or speed.

Manufacturer reference bands were not used for comparison because their source population and acquisition protocol are undisclosed and the printed bands are internally inconsistent. The propulsion index is a proprietary composite reported in arbitrary units; its calculation, minimal detectable change and validated post-TKA interpretive threshold are not published. It is therefore treated as a device-derived numerical observation and as nothing further. What may legitimately be taken from it is that, on this device’s own scale and at each of the three sessions, the value returned for the operated limb was lower than the value returned for the non-operated limb. What may not be taken from it is any statement about propulsion mechanics, push-off force, ground-reaction force, joint loading or plantarflexor function, none of which were measured. Neither can the size of the side-to-side difference be judged against any external benchmark, because no validated benchmark for this index exists.

### 2.5. Follow-Up and Outcomes

**Figure 1:**
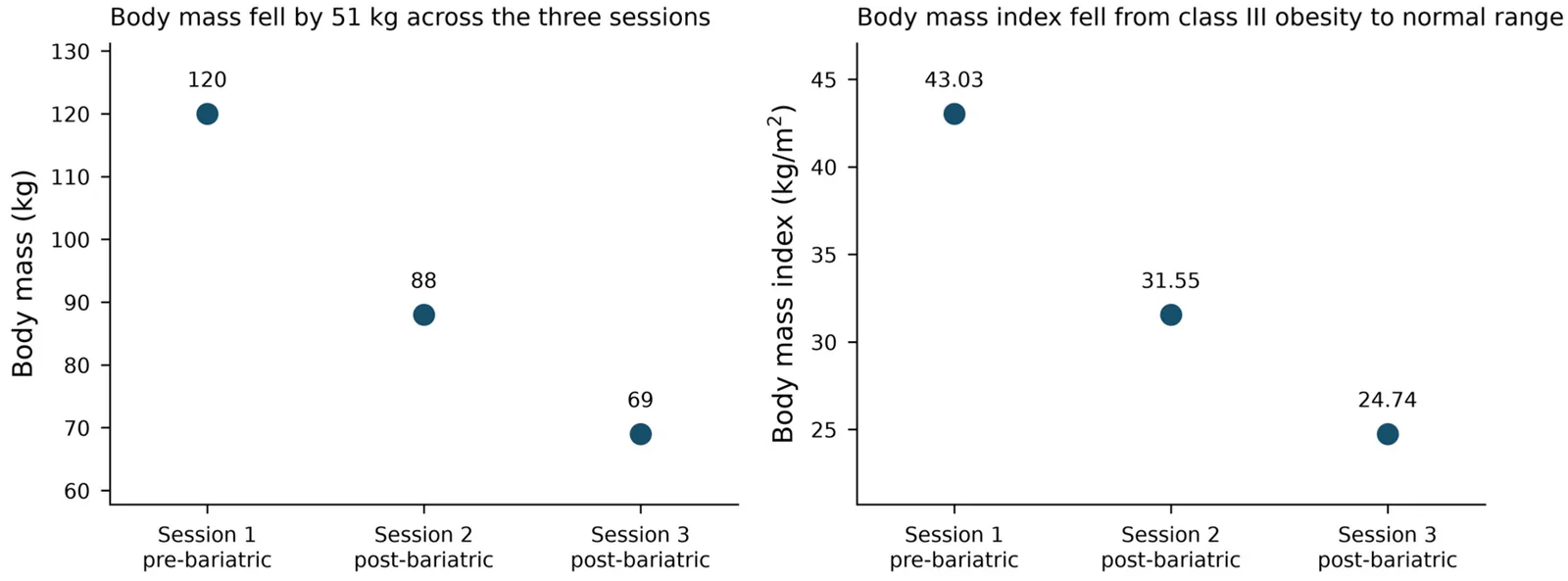
Body mass and body mass index across the three assessment sessions. Session 1 preceded bariatric surgery and sessions 2 and 3 followed it. Values are plotted as individual session observations without connecting lines. The exact date of bariatric surgery and the exact collection dates for sessions 1 and 2 were not recorded, so the intervals between the three points are unequal and partly unknown, and no continuous trajectory between sessions should be inferred from their relative positions. Body mass index was computed from the measured height of 1.67 m and is plotted to two decimal places.

Body mass was 120, 88 and 69 kg at sessions 1 to 3, corresponding to BMI values of 43.03, 31.55 and 24.74 kg/m^2^ (Table 1; Figure 1). The 120 kg and 69 kg values were recorded to the nearest kilogram in the source documentation and instrument reports, respectively; the 88 kg value was supplied by the treating team and was not present in the electronic gait export.

**Figure 2:**
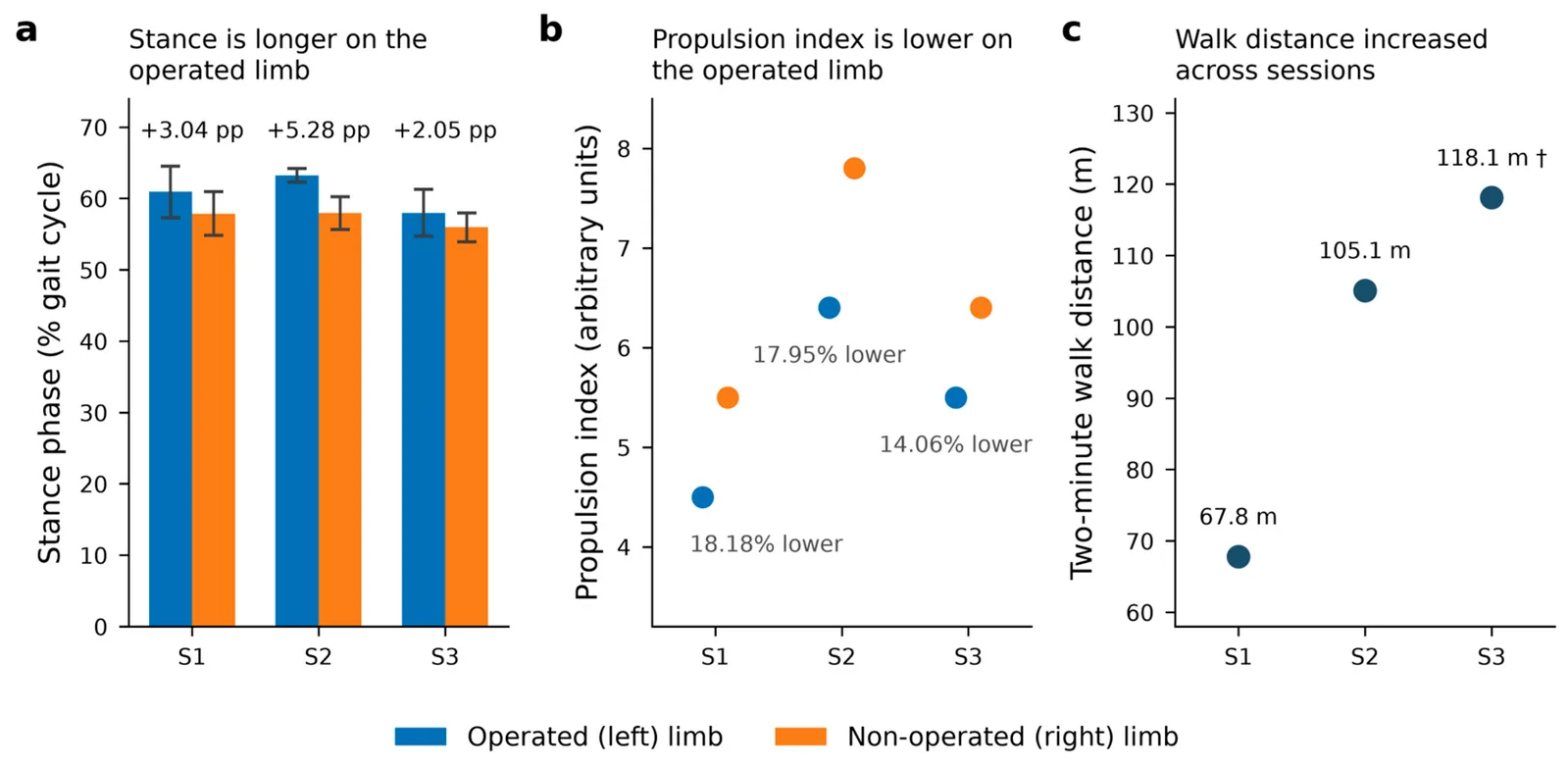
Interlimb measures and 2MWT distance by session. (**a**) Stance-phase duration for the operated and non-operated limbs; (**b**) the proprietary propulsion index; (**c**) 2MWT distance. Values are plotted as individual session observations without connecting lines, because the intervals between sessions are unequal and partly unknown and no continuous change between sessions should be inferred. Error bars in panel (**a**) are the standard deviation of the steps retained by the software within one walking trial. They quantify how much stance duration varied from step to step inside that single trial. They are not standard errors, not confidence intervals, and not an index of measurement reliability, repeatability or test-retest agreement, and they should not be used to judge whether two sessions differ. Step counts were 10, 9 and 5 on the operated limb and 10, 10 and 4 on the non-operated limb at sessions 1 to 3, so the session-3 dispersion is estimated from very few steps. No error bars are shown in panel (**b**) because the propulsion index is reported by the device as a single composite value per limb per session, with no step-level distribution, no published calculation and no validated post-TKA interpretive threshold. † The session-3 walk test was launched in the software’s six-minute module and terminated at 1 min 58 s; the plotted value is the distance accumulated over 118 s, without adjustment to 120 s.

2MWT distance was 67.8, 105.1 and 118.1 m across the three sessions (Figure 2), the session-3 value having been accumulated over 118 s rather than 120 s. The software reported 10, 11 and 12 completed course lengths with no rest intervals. These values are reported as repeated descriptive observations from one trial per session; no inferential statistics and no minimal-detectable-change interpretation were applied.

Limb-specific spatiotemporal values are shown in Table 2. Stance duration was longer on the operated left limb than on the non-operated right limb at each session: 60.91 ± 3.61% versus 57.87 ± 3.07% of the gait cycle at session 1, 63.23 ± 0.94% versus 57.95 ± 2.30% at session 2, and 57.98 ± 3.27% versus 55.93 ± 2.05% at session 3. The corresponding operated-minus-non-operated differences were 3.04, 5.28 and 2.05 percentage points (Figure 2). The proprietary propulsion index was also numerically lower on the operated side at each session (4.5 versus 5.5, 6.4 versus 7.8, and 5.5 versus 6.4 arbitrary units), equivalent to relative differences of 18.18%, 17.95% and 14.06%. The direction of both differences was the same at every session; their magnitude was not. The stance difference rose from session 1 to session 2 and then fell below its session-1 value, and the relative difference in the propulsion index was smallest at session 3. These are three separate observations that share a direction. They do not form a trend, they do not establish measurement stability, and they are not evidence of a persistent deficit.

Straight-line instrumented gait speed and metric stride length were computed at session 1 only (0.97 ± 0.06 m/s; operated-limb stride length 1.16 ± 0.07 m, non-operated limb 1.17 ± 0.06 m). At sessions 2 and 3 those fields were exported as zero by the software, which denotes a quantity not computed rather than absent movement; they are therefore reported as not computed. Pelvic kinematics were available at session 3 only and were not used for longitudinal comparison in the primary report.

Valid TUG total durations were available for sessions 1 and 2 (13.41 and 14.95 s). The available source documentation did not contain a valid session-3 TUG total; no session-3 TUG total or phase-level measurement is therefore reported or interpreted (Table 3).

## 3. Discussion

Across the three assessments, body mass decreased by 51 kg and 2MWT distance increased by 50.30 m. At the same assessments the operated limb showed longer stance duration than the non-operated limb by 3.04, 5.28 and 2.05 percentage points, and the proprietary propulsion index was numerically lower on the operated side. The appropriate interpretation is limited to repeated observations within one patient. Because each session contained one trial, and the third session retained only five operated-limb and four non-operated-limb steps, these data cannot establish a stable persistent gait deficit or a clinically meaningful change.

The increase in walking distance cannot be attributed to the reduction in body mass. Weight loss was one of several things that changed between the first and the last assessment. The interval since arthroplasty extended from approximately 31 to 67 months; postoperative rehabilitation was delivered after both procedures; continued natural recovery cannot be excluded; and the patient performed the same battery of tests on three occasions, so familiarisation with the protocol is also a candidate explanation. Unrecorded clinical factors may have contributed as well. None of these influences can be separated in a single uncontrolled case, and the title, Abstract and Discussion accordingly describe the change in walking distance as occurring across the period of weight loss and not as a consequence of it. The functional outcomes also did not all move in the same direction. The TUG total was numerically longer at session 2 than at session 1, at 14.95 versus 13.41 s, which is opposite in direction to the change in walking distance over the same interval. With one trial per session and no estimate of within-session variability for this test, a difference of 1.54 s carries no interpretive weight, and it is noted here only to show that overall functional performance did not change uniformly across measures.

The observations are nevertheless clinically informative, because whole-task distance and limb-specific spatiotemporal variables summarise different aspects of walking. Prior TKA studies report limb-to-limb differences in stance and propulsive performance after surgery [7,8,9,10], while bariatric-surgery studies report changes in gait and walking performance after major weight loss [13,14,15,16]. Interlimb asymmetry has also been related to body composition at population level, with asymmetry indices differing between adolescents with and without obesity [27], which places the present single-patient observation within a broader empirical pattern rather than leaving it isolated. In this patient an increase in 2MWT distance occurred alongside side-to-side differences in stance duration at each session. This co-occurrence does not demonstrate that the two measures changed independently, nor does it identify the mechanism responsible for either observation.

The propulsion index warrants particular caution. It is a proprietary composite with no published calculation method and no validated post-TKA threshold, and no kinetic, force-plate, electromyographic or strength measurement was obtained in this case. A lower value on the operated side is a number returned by a commercial algorithm. It is not evidence of reduced joint loading, diminished ground-reaction force or plantarflexor weakness, and it should not be read as a change in propulsion mechanics. The index is retained only as a descriptive device output, because it showed the same direction of side-to-side difference at each session. Before an asymmetry index of this kind can be interpreted at all, its reliability, sensitivity and minimal detectable change need to be established in the population in which it is to be used, as has been done for purpose-built asymmetry tests in other settings [28]. For the present index, these properties are unpublished and manufacturer thresholds cannot substitute for them.

### Limitations

This is a single uncontrolled case with three assessments and no comparison condition. Weight loss, time since arthroplasty, rehabilitation exposure, natural recovery and repeated exposure to the tests changed together and cannot be separated. Only one pre-bariatric assessment was available, so no pre-bariatric variability can be estimated. Important clinical covariates were not recorded, including comorbidities, medication, radiographic status, range of motion, strength and rehabilitation dose or adherence. Exact dates were unavailable for the first two assessments and for the bariatric procedure, so neither the rate of weight loss nor its temporal pattern relative to each assessment can be determined. It is not known how much of the 51 kg reduction had occurred by session 2, nor how recently the remainder had occurred before session 3, and weight change may therefore have been concentrated in an interval that no assessment brackets. WOMAC was collected only at the first session. Each test was performed once per session, so no test-retest assessment was possible, and standard deviations reflect within-trial step variability only. The third-session straight-line gait estimates were based on five operated-limb and four non-operated-limb steps. The session-3 walk test was terminated at 1 min 58 s rather than at 120 s. No adjustment was applied, but the 2 s shortfall is a minor protocol deviation that makes the session-3 distance not strictly comparable with the other two. Processing settings were incompletely documented. The two outputs that carry the main observations of this report, the interlimb stance difference and the propulsion index, have no published reliability data, and what a follow-up study would need to supply can be specified precisely: intraclass correlation coefficients for within-session and between-session agreement, and the minimal detectable change of each measure, both estimated from same-day repeated trials in the authors’ own post-arthroplasty population rather than inferred from validation performed elsewhere or from unpublished manufacturer thresholds. Pelvic output was available at one session only, and no kinetic or loading measurement was obtained.

## 4. Conclusions

In this single case, 2MWT distance increased across three assessments, spanning a 51 kg reduction in body mass, while operated-versus-non-operated differences in stance duration and a proprietary propulsion index were present at each assessment. These findings are descriptive and do not establish persistence, mechanical loading asymmetry, clinical importance or causation. The contribution of this report is hypothesis-generating. It shows that whole-task and limb-specific gait measures can give non-equivalent descriptions of the same patient at the same assessment, and it identifies what would be required to test that observation properly. Prospective studies should use repeated trials within each session, an analysed step count sufficient for stable spatiotemporal and variability estimates, transparent and published algorithms, and validated kinetic or limb-specific outcomes reported alongside whole-task performance.

## Figures and Tables

**Table 1:** Reconstructed clinical and assessment timeline.

Event	Time Relative to TKA	Body Mass (kg)	BMI (kg/m^2^)	Notes
Left total knee arthroplasty	Month 0	Not recorded	Not recorded	End-stage osteoarthritis; subvastus approach; rotating-platform implant
Session 1 instrumented assessment	~31 months	120	43.03	Pre-bariatric; WOMAC total 12/96
Roux-en-Y gastric bypass	Between sessions 1 and 2	Not recorded	Not recorded	Exact date not recorded
Session 2 instrumented assessment	Following calendar year; exact interval not recorded	88	31.55	Post-bariatric; body mass supplied by treating team
Session 3 instrumented assessment	~67 months	69	24.74	Post-bariatric; timing fixed by instrument reports

Time relative to TKA is expressed from left total knee arthroplasty at month 0. Exact dates for sessions 1 and 2 and for the bariatric procedure were not recorded, so the rate and temporal pattern of weight loss relative to each assessment cannot be determined. Body mass index was computed by the authors from the measured height of 1.67 m and is reported to two decimal places; body mass is reported as recorded, to the nearest kilogram. BMI: body mass index; TKA: total knee arthroplasty; WOMAC: Western Ontario and McMaster Universities Osteoarthritis Index.

**Table 2:** Instrumented straight-line gait variables by session and limb.

Variable	Session 1	Session 2	Session 3
Analysis duration (s)	29.4	26.3	30.2
Steps analysed, operated (left) limb (n)	10	9	5
Steps analysed, non-operated (right) limb (n)	10	10	4
Cadence (steps/min)	101.54 ± 15.11	93.87 ± 5.54	96.66 ± 8.80
Straight-line instrumented gait speed (m/s)	0.97 ± 0.06	Not computed	Not computed
Gait-cycle duration, operated limb (s)	1.21 ± 0.02	1.28 ± 0.05	1.27 ± 0.06
Gait-cycle duration, non-operated limb (s)	1.20 ± 0.10	1.30 ± 0.03	1.25 ± 0.08
Stance, operated limb (% cycle)	60.91 ± 3.61	63.23 ± 0.94	57.98 ± 3.27
Stance, non-operated limb (% cycle)	57.87 ± 3.07	57.95 ± 2.30	55.93 ± 2.05
Operated-minus-non-operated stance difference (percentage points)	3.04	5.28	2.05
Swing, operated limb (% cycle)	39.09 ± 3.61	36.77 ± 0.94	42.02 ± 3.27
Swing, non-operated limb (% cycle)	42.13 ± 3.07	42.05 ± 2.30	44.07 ± 2.05
Single support, operated limb (% cycle)	42.10 ± 4.20	37.47 ± 1.38	45.93 ± 4.51
Single support, non-operated limb (% cycle)	39.04 ± 2.60	42.77 ± 3.97	42.90 ± 2.00
Double support, operated limb (% cycle)	12.73 ± 3.52	12.57 ± 2.95	6.55 ± 0.75
Double support, non-operated limb (% cycle)	5.70 ± 1.40	7.79 ± 0.89	5.78 ± 0.90
Stride length, operated limb (m)	1.16 ± 0.07	Not computed	Not computed
Stride length, non-operated limb (m)	1.17 ± 0.06	Not computed	Not computed
Propulsion index, operated limb (a.u.)	4.5	6.4	5.5
Propulsion index, non-operated limb (a.u.)	5.5	7.8	6.4
Relative propulsion-index difference (%)	18.18	17.95	14.06

Values are mean ± standard deviation of the steps retained by the software within one walking trial. Standard deviations represent within-trial step-to-step dispersion and not measurement reliability. Session-3 values are derived from five operated-limb and four non-operated-limb steps and are correspondingly unstable. ‘Not computed’ denotes a field exported as zero by the software and not a measured value of zero. The propulsion index is a proprietary composite reported in arbitrary units without a published calculation or a validated post-TKA threshold; it is a device-derived numerical observation and is not a measure of push-off force, ground-reaction force or joint loading. Manufacturer reference bands are not presented because their source population and acquisition protocol are undisclosed and the printed bands are internally inconsistent. Differences reported in this table were computed by the authors from the values above and are given to two decimal places. a.u.: arbitrary units.

**Table 3:** Functional test outcomes, anthropometry and patient-reported outcome by session.

Measure	Session 1	Session 2	Session 3
Two-minute walk test distance (m)	67.8	105.1	118.1 †
Walk-test duration (s)	120	120	118 †
Course lengths completed (n)	10	11	12
Rest intervals taken (n)	0	0	0
Vendor-reported steady-state cadence (steps/min) ‡	91.6	101.1	104.1
Vendor-reported steady-state stride length (m) ‡	0.94	1.31	1.46
Timed Up and Go total (s)	13.41	14.95	Not recorded
WOMAC total (0–96)	12	Not administered	Not administered
Body mass (kg)	120	88	69
BMI (kg/m^2^)	43.03	31.55	24.74

† The walk test was administered as a two-minute walk at all sessions. At session 3, it was launched in the software’s six-minute module and terminated at 1 min 58 s, so the distance of 118.1 m was accumulated over 118 s rather than 120 s. No adjustment, pro-rating or extrapolation was applied, and the session-3 distance is therefore not strictly comparable with sessions 1 and 2. ‡ Cadence and stride length are per-step outputs of the device’s gait-event detection and describe the straight-line, steady-state portions of the course. Their product exceeds the measured walk-test distance by 27.00%, 26.01% and 26.55% at sessions 1 to 3, which is consistent with the exclusion of turning and non-steady-state intervals; they are therefore not used to reconstruct walk-test distance or speed. The proprietary vendor-derived walk-test speed output is not tabulated at all, because it is presented by the manufacturer as a whole-test quantity yet cannot be reconciled with distance divided by total test duration, and its calculation is undisclosed. Course lengths are software-reported path completions and not independent trials. The available source documentation did not contain a valid session-3 Timed Up and Go result, so no session-3 total or phase-level Timed Up and Go measurements are reported. BMI: body mass index; WOMAC: Western Ontario and McMaster Universities Osteoarthritis Index.

## Data Availability

The original data presented in this study are available on reasonable request from the corresponding author, subject to the privacy and de-identification conditions under which they were released. The data are not publicly available due to privacy concerns.

## Supplementary Materials

The following supporting information can be downloaded at: https://www.mdpi.com/article/10.3390/reports9040345/s1, Supplementary S1: Acquisition and Processing Protocol; Supplementary S2: provenance and interpretive status of proprietary device outputs; Supplementary S3: source-availability reconciliation and de-identification notes; Supplementary S4: the completed CARE checklist.

## Abbreviations

The following abbreviations are used in this manuscript:

2MWT	two-minute walk test
BMI	body mass index
CARE	CAse REport
IMU	inertial measurement unit
TKA	total knee arthroplasty
TUG	Timed Up and Go
WOMAC	Western Ontario and McMaster Universities Osteoarthritis Index
