# Supplementary material for: Walking Distance and Interlimb Gait Differences Across 51 kg of Weight Loss After Total Knee Arthroplasty: A Three-Session Wearable Sensor Case Report
Source: Reports (MDPI). 2026 Oct 12;9(4):345. doi: 10.3390/reports9040345 (PMC13692334; doi:10.3390/reports9040345)
Supplement: Supplementary file 1 [file reports-09-00345-s001.zip › Supplementary S1.pdf]

Supplementary S1: Acquisition and Processing Protocol

Supplementary material to: Walking Distance and Interlimb Gait Differences Across 51 kg of Weight Loss After Total Knee Arthroplasty: A Three-Session Wearable Sensor Case Report

1. Scope

This file sets out the acquisition and processing protocol for the three instrumented assessments reported in the main text. It records the procedure as it was actually carried out and documents, item by item, which elements of the processing chain could be established from the archived instrument reports and which could not. Nothing in this file has been reconstructed retrospectively; where information was absent from the source documentation it is stated as not recorded.

2. Instrumentation

- Sensor: a single wireless inertial measurement unit (IMU), G-Walk (BTS Bioengineering, Garbagnate Milanese, Italy).
- Placement: over the L5 spinous process, secured with the manufacturer's elastic belt.
- Sampling frequency: 100 Hz.
- Analysis software: the manufacturer's G-Studio software.
- Operator: the same assessor at all three sessions.
- Software and firmware version: not recorded. The version strings were not printed on the archived instrument reports and were not recorded separately at the time of assessment.

3. Session structure

Each session comprised one straight-line instrumented walk, one Timed Up and Go (TUG) test and one walk test, in that order, with no repetition of any test. No rest intervals were taken during the walk test. A Western Ontario and McMaster Universities Osteoarthritis Index (WOMAC) questionnaire was administered at session 1 only.

Element	Session 1	Session 2	Session 3
Straight-line walk analysis duration (s)	29.4	26.3	30.2
Steps retained, operated (left) limb (n)	10	9	5
Steps retained, non-operated (right) limb (n)	10	10	4
Walk-test module used	Two-minute walk	Two-minute walk	Six-minute module, stopped at 1 min 58 s
Walk-test duration (s)	120	120	118
Trials per test (n)	1	1	1
Rest intervals during walk test (n)	0	0	0
TUG trials (n)	1	1	1

WOMAC administered	Yes	No	No

4. Walk-test administration and the session-3 deviation

The walk test was administered as a two-minute walk test (2MWT) at all three sessions. At session 3 the operator launched the protocol in the software's six-minute module and terminated it at 1 min 58 s. The distance reported for that session, 118.1 m, is therefore the value accumulated over 118 s as exported by the software. No adjustment, pro-rating or extrapolation to 120 s was applied at any stage of the analysis or reporting. For reference only, simple pro-rating of 118.1 m from 118 s to 120 s would give 120.10 m; that figure is not used anywhere in the report. The session-3 distance is consequently derived from 2 s less walking than sessions 1 and 2 and is flagged as such in Table 3 and in the legend of Figure 2 of the main text, and listed as a protocol limitation.

5. Walking course

The length of the walking course, the manner in which turns were performed, and whether an identical course configuration was used at all three sessions were not recorded in the source documentation and cannot be verified retrospectively. The count of completed course lengths reported by the software (10, 11 and 12 at sessions 1 to 3) is an internal device output from which the course geometry cannot be recovered. Comparisons of walk-test distance across sessions should be read with this uncertainty in mind.

6. Signal processing: what was fixed and what is undisclosed

The acquisition used the vendor default protocols for the Walk, TUG and walk-test modules. The following statements describe the processing as performed:

- No user-configurable processing setting was altered at any session.
- The automatic segmentation produced by the software was accepted exactly as exported.
- No trial was rejected, repeated or re-run, and no manual editing of gait events was performed.
- No post-processing, smoothing or filtering was applied by the investigators outside the software.

The following elements of the processing chain are neither user-accessible nor published by the manufacturer, and therefore cannot be specified:

- Filter type and cut-off frequencies.
- Gait-event detection rule.
- Trial-rejection criteria applied internally by the software.
- Acceleration and deceleration exclusion windows.
- The calculation underlying each proprietary composite output (see File S2).

Exact reproduction of the signal processing is therefore not possible. The values reported in the main text should be read as the output of one commercial implementation rather than of a transparent algorithm. Independent validation has been reported for several spatiotemporal outputs of trunk-mounted systems in other populations, but that evidence does not validate the proprietary composite indices used by this device after total knee arthroplasty.

7. Numerical convention

Values computed by the authors, including body mass index, percentage-point differences and relative differences, are reported to two decimal places throughout the main text, tables and figures. Values exported directly by the instrument are reported at the precision at which the software exported them and are not padded to a precision the device did not provide. Body mass index was computed from the measured height of 1.67 m.

8. Statistical approach

No inferential statistics were applied at any stage. This was a design decision rather than an omission. With one patient, one trial per session and steps drawn from within that single trial, the repeated observations do not constitute an independent sampling distribution; a p value or confidence interval computed from them would describe dispersion among the steps of one walk while appearing to describe the precision of a population estimate. Standard deviations reported in Table 2 and plotted in Figure 2 of the main text are the dispersion of the steps retained by the software within a single trial. They are not standard errors, not confidence intervals and not an index of measurement reliability, repeatability or test-retest agreement. No minimal-detectable-change interpretation was applied to any variable.
