# Supplementary material for: Walking Distance and Interlimb Gait Differences Across 51 kg of Weight Loss After Total Knee Arthroplasty: A Three-Session Wearable Sensor Case Report
Source: Reports (MDPI). 2026 Oct 12;9(4):345. doi: 10.3390/reports9040345 (PMC13692334; doi:10.3390/reports9040345)
Supplement: Supplementary file 1 [file reports-09-00345-s001.zip › Supplementary S2.pdf]

Supplementary S2: Provenance and Interpretive Status of Proprietary Device Outputs

Supplementary material to: Walking Distance and Interlimb Gait Differences Across 51 kg of Weight Loss After Total Knee Arthroplasty: A Three-Session Wearable Sensor Case Report

1. Purpose

The G-Walk system exports a mixture of directly measured spatiotemporal quantities and vendor-computed composite indices. The two classes do not carry the same evidential weight, and this file records, for every output encountered, where the value came from, whether its calculation is published, and how it was used in the main text. The distinction matters because the two observations the report is built on, the interlimb stance difference and the propulsion index, fall on opposite sides of it.

2. Status of each device output

Output	Class	Published calculation	Use in this report
Cadence (straight-line walk)	Directly derived per-step quantity	Not published; derived from detected gait events	Reported in Table 2
Gait-cycle duration, per limb	Directly derived per-step quantity	Not published; derived from detected gait events	Reported in Table 2
Stance, swing, single support, double support (% cycle)	Directly derived per-step quantities	Not published; derived from detected gait events	Reported in Table 2; stance carries one of the two main observations
Straight-line instrumented gait speed	Directly derived quantity	Not published	Session 1 only; exported as zero at sessions 2 and 3, reported as not computed
Stride length (straight-line walk)	Directly derived quantity	Not published	Session 1 only; exported as zero at sessions 2 and 3, reported as not computed
Propulsion index	Proprietary composite, arbitrary units	Not published; no minimal detectable change, no validated post-TKA threshold	Reported descriptively only; see section 4 below
Walk-test distance	Directly measured whole-test quantity	Not applicable	Primary walk-test outcome; Table 3 and Figure 2
Walk-test cadence and stride length	Per-step quantities restricted to steady-	Not published	Reported in Table 3, labelled as steady-state

	state intervals		descriptors; see section 3
Walk-test speed	Proprietary composite presented as a whole-test quantity	Not published; cannot be reconciled with distance divided by duration	Excluded from the main text; see section 3
Course lengths completed	Internal device count	Not published	Reported in Table 3; course geometry cannot be recovered from it
Manufacturer reference bands	Vendor normative bands	Source population and acquisition protocol undisclosed; printed bands internally inconsistent	Not used for any comparison
Pelvic kinematics	Directly derived quantities	Not published	Available at session 3 only; not used for longitudinal comparison
TUG total and phase durations	Directly derived quantities	Not published	Totals reported for sessions 1 and 2; no valid session-3 result in the source documentation

3. Why walk-test speed was excluded while cadence and stride length were retained

The vendor's walk-test speed output is not equal to distance divided by total test duration, and the manufacturer does not disclose the calculation or whether turning and other non-steady-state intervals are excluded. Because it is offered as a whole-test quantity but cannot be reconciled with the whole test, it is not interpreted as conventional walking speed anywhere in the report.

Cadence and stride length are retained on a different basis. Both are direct per-step quantities produced by the same gait-event detection whose spatiotemporal accuracy has been examined independently in trunk-mounted systems. An internal check supports treating them as steady-state descriptors rather than whole-test quantities: multiplying the reported cadence by the reported stride length over the test duration implies a distance that exceeds the measured walk-test distance by a near-constant factor at all three sessions, across sessions whose measured distances differ by a factor of 1.7.

Quantity	Session 1	Session 2	Session 3
Vendor-reported steady-state cadence (steps/min)	91.6	101.1	104.1
Vendor-reported	0.94	1.31	1.46

steady-state stride length (m)			
Test duration (s)	120	120	118
Implied distance, cadence x stride length x duration (m)	86.10	132.44	149.45
Measured walk-test distance (m)	67.8	105.1	118.1
Excess of implied over measured (%)	27.00	26.01	26.55

This pattern is what would be expected if cadence and stride length describe the straight-line, steady-state portions of the course while excluding the turns and the accelerations and decelerations around them. That is internally coherent for per-step descriptors, but it is not reconcilable with a whole-test average, which necessarily includes the turns. The two variables are therefore reported in Table 3 as descriptors of detected steady-state walking and are not used to reconstruct walk-test distance or speed.

4. Interpretive status of the propulsion index

The propulsion index is a proprietary composite reported in arbitrary units. Its calculation, its minimal detectable change and any validated post-arthroplasty interpretive threshold are unpublished. It is treated throughout as a device-derived numerical observation and as nothing further.

- What may legitimately be taken from it: that, on this device's own scale and at each of the three sessions, the value returned for the operated limb was lower than the value returned for the non-operated limb.
- What may not be taken from it: any statement about propulsion mechanics, push-off force, ground-reaction force, joint loading or plantarflexor function. None of these were measured, and no kinetic, force-plate, electromyographic or strength data were obtained.
- What cannot be judged at all: the clinical size of the side-to-side difference, because no validated external benchmark for this index exists.

The index is reported by the device as a single composite value per limb per session, with no step-level distribution. This is why no error bars appear in the corresponding panel of Figure 2. Establishing the intraclass correlation coefficient and the minimal detectable change of this index in the authors' own post-arthroplasty population, from same-day repeated trials, is identified in the main text as a prerequisite for any future interpretation.
