# Supplementary material for: Walking Distance and Interlimb Gait Differences Across 51 kg of Weight Loss After Total Knee Arthroplasty: A Three-Session Wearable Sensor Case Report
Source: Reports (MDPI). 2026 Oct 12;9(4):345. doi: 10.3390/reports9040345 (PMC13692334; doi:10.3390/reports9040345)
Supplement: Supplementary file 1 [file reports-09-00345-s001.zip › Supplementary S3.pdf]

Supplementary S3: Source-Availability Reconciliation and De-identification Notes

Supplementary material to: Walking Distance and Interlimb Gait Differences Across 51 kg of Weight Loss After Total Knee Arthroplasty: A Three-Session Wearable Sensor Case Report

1. Purpose

This case was assembled from records collected during routine care and a previously approved institutional research protocol rather than from a prospective data-collection instrument. This file states, for every reported variable, which source it came from and at which sessions it was available, and lists the information that was absent from the source documentation. No value was inferred, imputed or reconstructed; absent items are recorded as not recorded.

2. Sources used

- Source documentation: the clinical records held by the treating service.
- Instrument reports: the archived G-Studio reports exported at each session.
- Treating team: a value supplied directly by the clinicians responsible for the patient and not present in the electronic gait export.

3. Variable-by-variable reconciliation

Variable	Source	S1	S2	S3
Body mass	Source documentation (120 kg); treating team (88 kg); instrument report (69 kg)	Yes	Yes	Yes
Height	Source documentation	Yes	-	-
Body mass index	Computed by the authors from height and body mass	Yes	Yes	Yes
Straight-line spatiotemporal variables	Instrument reports	Yes	Yes	Yes
Straight-line gait speed and stride length	Instrument reports	Yes	Exported as zero	Exported as zero
Propulsion index	Instrument reports	Yes	Yes	Yes
Walk-test distance and duration	Instrument reports	Yes	Yes	Yes
Walk-test cadence	Instrument reports	Yes	Yes	Yes

and stride length				
Timed Up and Go total	Instrument reports	Yes	Yes	No valid result in the source documentation
Pelvic kinematics	Instrument reports	No	No	Yes
WOMAC	Source documentation	Yes	Not administered	Not administered
Session timing relative to arthroplasty	Instrument reports (session 3); source documentation (sessions 1 and 2, approximate)	Approximate	Interval not recorded	Yes

A field exported as zero by the software denotes a quantity the software did not compute, not a measured value of zero. Such fields are reported as not computed in Table 2 of the main text and were excluded from all comparisons.

4. Information absent from the source documentation

The following were sought and are not recorded. They are listed here in full so that the boundary of the available record is explicit, and they are acknowledged as limitations in the main text.

- Clinical: presenting symptoms, detailed physical examination, radiographic osteoarthritis grade, coronal deformity, range of motion, prosthesis alignment, radiographic status at follow-up, comorbidities, medication, nutritional status, smoking history and occupation.
- Interval clinical course: knee pain, functional complaints, postoperative complications and rehabilitation characteristics between sessions. The only interval information available is the session-1 WOMAC subscale scores (pain 1 of 20, stiffness 0 of 8, physical function 11 of 68) and the documented absence of an assistive device at every session.
- Surgical: implant manufacturer, model, bearing material, fixation, patellar management, operative time and alignment strategy.
- Rehabilitation: type, frequency, duration, adherence and associated adverse events, after both procedures.
- Timing: the exact collection dates of sessions 1 and 2 and the exact date of the Roux-en-Y gastric bypass. The interval between sessions 1 and 2, and the position of the bariatric procedure within that interval, are therefore indeterminate, and the rate and temporal pattern of weight loss relative to each assessment cannot be determined.
- Instrumentation: G-Studio software and firmware version strings, and the walking-course length and turning arrangement (see File S1).

5. De-identification

The data were released to the authors under de-identification conditions set by the host institution. The following measures were applied and constrain what can be reported:

- Age is given as a band ('a man in his early sixties') rather than as a value.
- No calendar dates appear anywhere in the report. All timing is expressed as months relative to the arthroplasty, which is set at month 0.
- No name, record number, institutional identifier, image of the patient or other direct identifier appears in the manuscript, the figures or these files.
- A structured patient perspective was not collected at the time of assessment and could not be obtained retrospectively without breaching the conditions under which the data were released.

For the same reason the Data Availability Statement qualifies the release of de-identified source files and raw instrument reports as subject to these conditions. Written informed consent for publication of this case report and the accompanying anonymised data and figures was obtained from the patient.
