# Supplementary material for: Walking Distance and Interlimb Gait Differences Across 51 kg of Weight Loss After Total Knee Arthroplasty: A Three-Session Wearable Sensor Case Report
Source: Reports (MDPI). 2026 Oct 12;9(4):345. doi: 10.3390/reports9040345 (PMC13692334; doi:10.3390/reports9040345)
Supplement: Supplementary file 1 [file reports-09-00345-s001.zip › Supplementary S4.pdf]

Supplementary S4: Completed CARE Checklist

Supplementary material to: Walking Distance and Interlimb Gait Differences Across 51 kg of Weight Loss After Total Knee Arthroplasty: A Three-Session Wearable Sensor Case Report

The checklist below follows the 13-item CARE checklist (2013) cited as reference 22 of the main text. For each item the response is given as Yes, Partly, No or Not applicable, together with the location in the manuscript or, where an item is not reported, the reason. Because this case was assembled from records collected during routine care rather than from a prospective data-collection instrument, several items could not be completed; in every such case the absence is stated in the manuscript as not recorded rather than inferred, and the corresponding entry below names where that statement appears. File S3 lists the absent information in full.

**Item**	**Topic**	**Checklist item description**	**Reported**	**Location in the manuscript, or reason**
1	Title	The area of focus and 'case report' should appear in the title	Yes	Title. The title names the area of focus (walking distance and interlimb gait differences across 51 kg of weight loss after total knee arthroplasty) and ends with 'A Three-Session Wearable Sensor Case Report'.
2	Key Words	Two to five key words that identify topics covered in this case report	Partly	Keywords. Seven keywords are given rather than the two to five suggested by CARE, in order to cover both the clinical and the instrumentation topics of the report; 'case report' is included.
3a	Abstract	Introduction: what is unique	Yes	Abstract, Background and

		about this case and what does it add to the medical literature?		Clinical Significance. States that longitudinal observations combining major postoperative weight loss with limb-specific gait assessment after arthroplasty are uncommon.
3b	Abstract	The main symptoms of the patient and the important clinical findings	Partly	Abstract, Case Presentation. The important clinical findings are reported. Presenting symptoms were not recorded in the source documentation (Section 2.2) and are therefore not stated.
3c	Abstract	The main diagnoses, therapeutic interventions and outcomes	Yes	Abstract, Case Presentation. End-stage osteoarthritis of the left knee, total knee arthroplasty and subsequent Roux-en-Y gastric bypass, with the three-session outcomes.
3d	Abstract	Conclusion: what are the main 'take-away' lessons from this case?	Yes	Abstract, Conclusions. The findings are descriptive and hypothesis-generating and cannot be

				attributed to weight loss specifically.
4	Introduction	Briefly summarize why this case is unique, with medical literature references	Yes	Section 1, Introduction and Clinical Significance.
5a	Patient Information	De-identified demographic and other specific information about the patient	Yes	Section 2.2. Age is given as a band and no calendar dates appear anywhere, under the de-identification conditions described in File S3.
5b	Patient Information	Main concerns and symptoms of the patient	No	Not recorded. Section 2.2 states that presenting symptoms were not recorded. The only related information available is the session-1 WOMAC pain and stiffness subscale scores.
5c	Patient Information	Medical, family and psychosocial history, including relevant genetic information	No	Not recorded. Section 2.2 lists comorbidities, medication, nutritional status, smoking history and occupation as not recorded; no family or genetic information was available. Acknowledged in

				Limitations.
5d	Patient Information	Relevant past interventions and their outcomes	Partly	Section 2.3. Left total knee arthroplasty at month 0 through a subvastus approach with a rotating-platform implant, and a subsequent Roux-en-Y gastric bypass. Implant details, operative time and the exact bypass date were not recorded.
6	Clinical Findings	Describe the relevant physical examination and other significant clinical findings	Partly	Section 2.2. Independent ambulation without an assistive device at all three sessions is reported. Detailed physical examination, radiographic grade, coronal deformity, range of motion and prosthesis alignment were not recorded.
7	Timeline	Relevant data from this episode of care organized as a timeline (figure or table)	Yes	Table 1 and Figure 1. The footnote to Table 1 states that exact dates for sessions 1 and 2 and for the bariatric procedure were not recorded, so the rate and temporal pattern

				of weight loss cannot be determined.
8a	Diagnostic Assessment	Diagnostic testing (such as physical examination, laboratory testing, imaging, surveys)	Yes	Section 2.4. Instrumented gait analysis with a trunk-mounted inertial measurement unit, Timed Up and Go, two-minute walk test and the WOMAC questionnaire.
8b	Diagnostic Assessment	Diagnostic challenges (such as access to testing, financial, or cultural)	Yes	Section 2.4 and Limitations. The vendor's processing chain is undisclosed, the software and firmware versions were not recorded, the walking-course geometry was not recorded, and few steps were retained at session 3.
8c	Diagnostic Assessment	Diagnostic reasoning, including other diagnoses considered	Not applicable	The diagnosis of end-stage osteoarthritis of the left knee was established before the episode reported here (Section 2.2), and no differential diagnosis was undertaken within this report.
8d	Diagnostic Assessment	Prognostic characteristics	Not applicable	No prognostic staging system

		(such as staging in oncology) where applicable		applies to this presentation and no prognostic characteristics were recorded.
9a	Therapeutic Intervention	Types of intervention (such as pharmacologic, surgical, preventive, self-care)	Yes	Section 2.3. Surgical: left total knee arthroplasty and Roux-en-Y gastric bypass. Postoperative rehabilitation was prescribed after both procedures.
9b	Therapeutic Intervention	Administration of intervention (such as dosage, strength, duration)	No	Not recorded. Section 2.3 states that implant manufacturer, model, bearing material, fixation, patellar management, operative time and alignment strategy, and the type, frequency, duration and adherence of rehabilitation, were not recorded.
9c	Therapeutic Intervention	Changes in intervention, with rationale	Not applicable	No change of intervention occurred, or was recorded, within the observation period.
10a	Follow-up and Outcomes	Clinician- and patient-assessed outcomes, where appropriate	Partly	Sections 2.5, Tables 2 and 3. Clinician-assessed instrumented and functional outcomes are

				reported at all three sessions. The patient-assessed outcome (WOMAC) was available at session 1 only, so no longitudinal patient-reported change can be described.
10b	Follow-up and Outcomes	Important follow-up diagnostic and other test results	Yes	Tables 2 and 3, Figures 1 and 2. Radiographic follow-up was not recorded (Section 2.2).
10c	Follow-up and Outcomes	Intervention adherence and tolerability (how was this assessed?)	No	Not recorded. Rehabilitation adherence and tolerability were not documented (Section 2.3) and could not be assessed retrospectively.
10d	Follow-up and Outcomes	Adverse and unanticipated events	No	Not recorded. Section 2.3 states that adverse events associated with rehabilitation were not recorded. The one protocol deviation, termination of the session-3 walk test at 1 min 58 s, is reported in Section 2.4, in Table 3, in the legend of Figure 2 and in

				Limitations.
11a	Discussion	Discussion of the strengths and limitations of the management of this case	Yes	Section 3 and Limitations.
11b	Discussion	Discussion of the relevant medical literature	Yes	Section 3, with reference to post-arthroplasty interlimb differences and to gait change after major weight loss.
11c	Discussion	The rationale for conclusions, including assessment of cause and effect	Yes	Section 3 states that the increase in walking distance cannot be attributed to the reduction in body mass, and names the competing influences that changed over the same interval.
11d	Discussion	The main 'take-away' lessons of this case report	Yes	Section 4, Conclusions. The contribution is identified as hypothesis-generating, with the specific requirements for future study.
12	Patient Perspective	The patient should share their perspective on the treatment they received	No	Section 2.1. A structured patient perspective was not collected at the time of assessment and could not be obtained retrospectively without breaching

				the de-identification conditions under which the data were released.
13	Informed Consent	Did the patient give informed consent? Please provide if requested	Yes	Section 2.1 and the Informed Consent Statement. Written informed consent for publication of this case report and the accompanying anonymised data and figures was obtained from the patient and is available to the editor on request.

Of the 30 checklist entries, 16 are reported in full and 5 in part; 6 are not reported because the required information was absent from the source documentation, and 3 are not applicable to this presentation. No item was completed by retrospective reconstruction.
